# Morphological growth pattern of *Phanerochaete chrysosporium* cultivated on different *Miscanthus x giganteus* biomass fractions

**DOI:** 10.1186/s12866-021-02350-8

**Published:** 2021-11-17

**Authors:** Hassan Khalil, Estelle Legin, Bernard Kurek, Patrick Perre, Behnam Taidi

**Affiliations:** 1 LGPM, CentraleSupélec, SFR Condorcet FR CNRS 3417, Centre Européen de Biotechnologie et de Bioéconomie (CEBB), Université Paris-Saclay, 3 Rue des Rouges Terres, 51110 Pomacle, France; 2 Université de Reims Champagne-Ardenne, INRAE, FARE, UMR A 614, Chaire AFERE, 51097 Reims, France; 3 LGPM, CentraleSupélec, Université Paris-Saclay, 8-10 Rue Joliot-Curie, 91190 Gif-sur-Yvette, France

**Keywords:** *Miscanthus x giganteus*, Solid-state fermentation, *Phanerochaete chrysosporium*, Microscopy and image processing, Spore germination, Mycelial growth

## Abstract

**Background:**

Solid-state fermentation is a fungal culture technique used to produce compounds and products of industrial interest. The growth behaviour of filamentous fungi on solid media is challenging to study due to the intermixity of the substrate and the growing organism. Several strategies are available to measure indirectly the fungal biomass during the fermentation such as following the biochemical production of mycelium-specific components or microscopic observation. The microscopic observation of the development of the mycelium, on lignocellulosic substrate, has not been reported. In this study, we set up an experimental protocol based on microscopy and image processing through which we investigated the growth pattern of *Phanerochaete chrysosporium* on different *Miscanthus x giganteus* biomass fractions.

**Results:**

Object coalescence, the occupied surface area, and radial expansion of the colony were measured in time. The substrate was sterilized by autoclaving, which could be considered a type of pre-treatment. The fastest growth rate was measured on the unfractionated biomass, followed by the soluble fraction of the biomass, then the residual solid fractions. The growth rate on the different fractions of the substrate was additive, suggesting that both the solid and soluble fractions were used by the fungus. Based on the FTIR analysis, there were differences in composition between the solid and soluble fractions of the substrate, but the main components for growth were always present. We propose using this novel method for measuring the very initial fungal growth by following the variation of the number of objects over time. Once growth is established, the growth can be followed by measurement of the occupied surface by the mycelium.

**Conclusion:**

Our data showed that the growth was affected from the very beginning by the nature of the substrate. The most extensive colonization of the surface was observed with the unfractionated substrate containing both soluble and solid components. The methodology was practical and may be applied to investigate the growth of other fungi, including the influence of environmental parameters on the fungal growth.

**Supplementary Information:**

The online version contains supplementary material available at 10.1186/s12866-021-02350-8.

## Background

Fungal solid-state fermentation (SSF) is an aerobic culture system in which filamentous fungi are grown on the surface of and/or within substrate biomass, with adequate water activity and a sufficiently network that allows air penetration [1]. Examples of industrial applications of SSF include pre-treatment of agricultural by-products to produce simple sugars (e.g., glucose and xylose) [2], enzymes (e.g., cellulases, xylanases, ligninases) [3, 4], organic acids (e.g., citric acid) [5], pigments [6], flavours [7], and fine chemicals. In the food industry, SSF is used to manufacture food additives and to produce fermented foods such as ripened cheese (e.g., Roquefort) [8] and fermented rice (e.g., tapé, tempeh) [9, 10] and even to grow edible mushrooms (e.g., *Agaricus bisporus*) [11]. More recently, SSF has been proposed for the potential biodegradation of hazardous compounds (e.g., polychlorinated biphenyls PCBs [12], the bioremediation of nitrocellulose pollution [13, 14], and for the detoxification of agro-industrial waste (e.g., coffee pulp) [15].

In natural ecosystems, filamentous fungi degrade the lignocellulosic biomass that is otherwise difficult to decompose. Lignin, cellulose, and hemicelluloses are the major polymeric constituents of lignocellulosic materials [16], such as hardwoods, softwoods, and non-woody plants [17]. It is well known that lignocellulosic biomass can be used as a potential feedstock in biorefinery operations [18]. Among those biomass sources is *Miscanthus x giganteus* (hereafter referred to as miscanthus), a rhizomatous perennial grass [19, 20], with a high cellulose content (> 40% w/w) in its stalks and branches [17]. This plant is an excellent candidate to provide lignocellulosic bioenergy due to its low nutrient requirements and high productivity [21, 22]. According to France Miscanthus (www.france-miscanthus.org), the total cultivated surface of miscanthus in France was greater than 6000 ha in 2019, with a 10% annual increase in recent years [23]. The dry matter yields in France are 15 to 40 t/ha [22], which can be turned into combustible solid (pellets) [24], used as a lignocellulosic substrate to produce second-generation liquid biofuels through fermentation, biogas generation by anaerobic digestion, production of high quality animal bedding or even manufacture of packaging materials [25–28].

Among the three main groups of wood rot fungi (white, brown, and soft rot-fungi), white-rot fungi are capable of efficiently mineralizing lignin, and cellulose and hemicelluloses in woody biomass [29]. These three groups of fungi occupy different ecological niches, such as deciduous trees, agricultural crops, and plant roots [30]. *Phanerochaete chrysosporium* is a white-rot fungus, used frequently as an experimental organism. Its genome has been fully sequenced [31]. Its lignocellulolytic system has been extensively studied, which has led to the delineation of the main complex mechanisms of the plant biomass degradation processes [32–34].

*Phanerochaete chrysosporium* quickly colonizes wood, degrading the lignin but often leaving cellulose almost unaffected [35–38]. The fungus excretes multiple lignin and manganese peroxidases (LiPs and MnPs) to mineralize lignin, the most recalcitrant component in lignocellulosic biomass [37]. The ligninolytic activities occur principally after the primary growth phase, once nutrient-limitation is encountered by the organism [39]. The regulation and the expression of *P. chrysosporium* genes encoding ligninolytic enzymes have been reported on wood and defined medium (rich/poor medium in nitrogen/carbon) [40–42]. The fungus can efficiently depolymerize polysaccharides in plant biomass by using a battery of hydrolytic enzymes [43]. The *P. chrysosporium* genome contains a vast repertoire of cellulose-, hemicellulose-, polysaccharide-degrading enzymes [30, 31]. The cellulolytic-enzymes are constitutive and continue to be produced during the different phases of fungal growth and degradation of lignocellulosic biomass [44]. Among 35 fungal species studied for miscanthus degradation, *P. chrysosporium* showed the greatest degrading activity, with approximately 20% of biomass weight loss after 8 weeks [45].

The robustness of the SSF process, including the colonization of the substrate by the desired microorganisms, is a major technological issue [1, 46]. Spore inocula offer high repeatability in the cultivation process [47]. Generally, a sufficient concentration of spores allows for the rapid proliferation of fungal biomass, guarantees a significant production of metabolites, and reduces the risk of contamination [48]. Thus, the ability to monitor inoculation, germination, and initial growth is a prerequisite for accurate process control and efficient conversion of lignocellulosic substrate into desired products.

Measuring the growth of filamentous fungi is challenged by practical difficulties. The mycelium becomes attached to the substrate and could penetrate deeply, complicating any attempts to recover it and, therefore, to determine the fungal biomass by weight. Indirect methods are then alternative solutions for the estimation of the fungal biomass produced during solid-state fermentation. Cellular components such as glucosamine, ergosterol and nucleic acids are often used as indicators of biomass [49–51]. A correlation between mycelium dry weight and such biochemical markers must be first calibrated in liquid culture before being applied to biomass measurements in solid state environment [52–54].

Bio-imaging is an ever-advancing technology to characterize and quantify the morphology of fungi during growth stages and under different culture conditions [55–57]. Various microscopic techniques are used to quantify relative growth and morphology. Field emission gun scanning electron microscopy (FEG-SEM) is a 3D ultrastructural approach for characterizing the size and shape of spores and the morphology of filaments in solid systems. Despite the large depth of field offered by this method, it relies on destructive fixation and dehydration, resulting in the inactivation of living samples, preventing continuous observation over time [58]. Laser scanning confocal microscopy (LSCM), a non-destructive 3D observation method, has been used for morphological characterization and quantification of mycelial growth [59]. The advantage of this technique is the level of detail of obtained data, but the use of fluorochromes to visualize the mycelial biomass weakens the approach.

In 2D observations, specific key data are inaccessible, such as tip extension rate, branch angle, branching length distribution, hyphal fusion, and the volume of fungal biomass as a number of voxels. For 3D high-resolution observation, the very slow point-by-point scanning rates result in long acquisition times that are not only inconvenient but even impossible to apply without influencing the growth of the organism. Using dyes for reducing scan time or increasing resolution can also affect the organism under study [60].

Non-destructive 2D observation of filamentous fungi in Petri dish cultures can be performed using a flat-bed scanner but resulting in low-resolution images [55]. Spore germination and mycelium formation of *Penicillium expansum* and *Aspergillus niger* have been observed on agar for extended periods using time-lapse photography [61]. Researchers have often monitored the growth of one mycelial colony originating from the germination of single spore without accounting for the realistic event in SSF in which a large population of spores is inoculated into the substrate [56, 62, 63]. Experimental data reported [61] heterogeneous germination times for individual spores even though they were all treated under the same conditions. This heterogeneity can be corrected by following a large number of spores. The larger the population the more realistic would be the observation. Data on the behaviour of a large number of colonies originating from single spores during the colonization of a natural substrate is still lacking.

Image analysis has been used to study the impact of environmental conditions on the growth of filamentous fungi at different stages. Some studies examined the germination of spores, others the growth dynamics starting from a mycelia fragment, and rarely from germination to mycelium development [55, 59, 61, 64]. Generally, the primary focus has been on temperature, relative humidity, aeration, synthetic substrate composition, and/or nutrient concentration [55, 61, 65]. To our knowledge, all the techniques used to follow growth on a natural substrate are destructive with two notable exceptions based on the same technique: stereomicroscopy with time-lapse digital imaging was used to follow *Aspergillus niger* mycelial growth on wheat straw [66, 67].

The first hours of fermentation on solid substrate determine the future success of the culture [68, 69]. In non-axenic SSF, other microorganisms (such as bacteria) besides the fungus could invade the medium [70, 71]. These organisms can lower process productivity and specific metabolite production if their growth is faster than that of the selected fungus [71, 72], so the desired species must outcompete other species that may be present.

The timely germination of fungal spores, the rapid elongation of the germ tubes, and the generation of new branches are the principal factors in the successful development of mycelium in SSF. The present study aims to explore the germination and growth characteristics of *P. chrysosporium* on a natural substrate during the first 52 h of fermentation. An experimental system was developed to investigate the growth characteristics of *P. chrysosporium* on different fractions of miscanthus non-invasively. How *P. chrysosporium* consumes the different components (soluble and solid) of miscanthus was monitored and provided insight into the way this organism attacks its substrate.

The visualisation method used in this study was not destructive and didn’t use the addition of fluorochrome. The images were further treated with bespoke image analysis to extract objective and quantitative information.

## Results

The growth of *P. chrysosporium* on solid media containing different fractions of miscanthus was followed by non-destructive microscopic observation. Two cultures were used as reference cultures: culture A which was devoid of any added miscanthus and culture B that incorporated the whole non-fractionated miscanthus. Culture A consisted of medium A containing only agar and the superimposed nitrocellulose membrane. The experiment was terminated for all the cultures when the fastest-growing culture started to reach the maximal image size (24 mm wide).

**Fig. 1:**
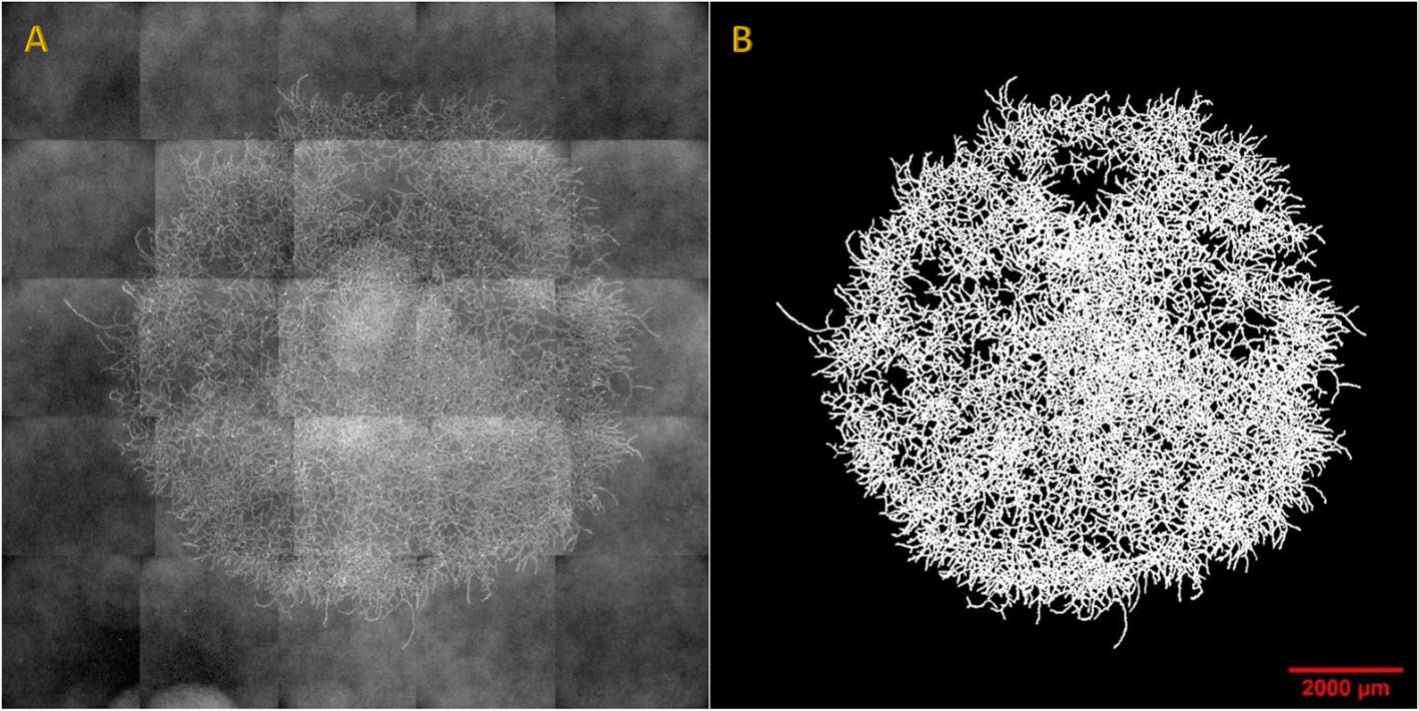
Example of acquired and processed images of a *P. chrysosporium* colony. The image acquired after 52 h of growth on agar medium is on the left (**A**). The processed image after image treatment operations (as described in the text) is on the right (**B**). Red scale bar = 2000 μm

The experimental apparatus and data processing methods allowed for successive observation of *P. chrysosporium* growth on the different miscanthus biomass fractions incorporated in agar. An example of the image produced from a 52 h growth of *P. chrysosporium* on agar medium without any fraction of miscanthus is shown in Fig. 1. The left image is composed of 25 single overlapping images (tiles).

The tiles were separated and reassembled into one image. Images with good black/white contrast were obtained, allowing for accurate quantification of the surface occupied by the fungus. Growth measurements (occupied area, colony diameter, germination rate, the number of objects) were determined from the processed images.

**Fig. 2:**
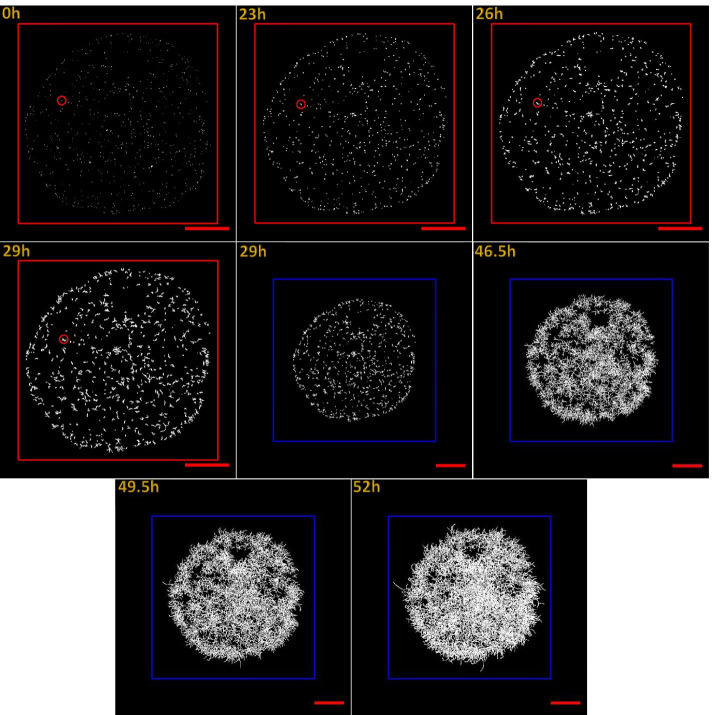
Germination and growth of *P. chrysosporium* on agar (defined as negative control – substrate A). Monitoring was performed at 50X magnification. Images show the growth at time 0, 23, 26, 29, 46.5, 49.5, and 52 h. A scale-shift of the image is indicated by the change in the colour of the drawn squares from red to blue. The sides of the red square measure 9000 μm. The length of the sides of the blue squares was 11,000 μm, and the image size is 12,288-pixel × 12,288-pixel. The red circles show the position of a single spore over time. At 23 h, the spore was swollen. At 26 h, a germ tube was formed. A microcolony was observed at 29 h, where branching started. The final colony can be seen at 52 h. The red scale bars measure 2000 μm

**Fig. 3:**
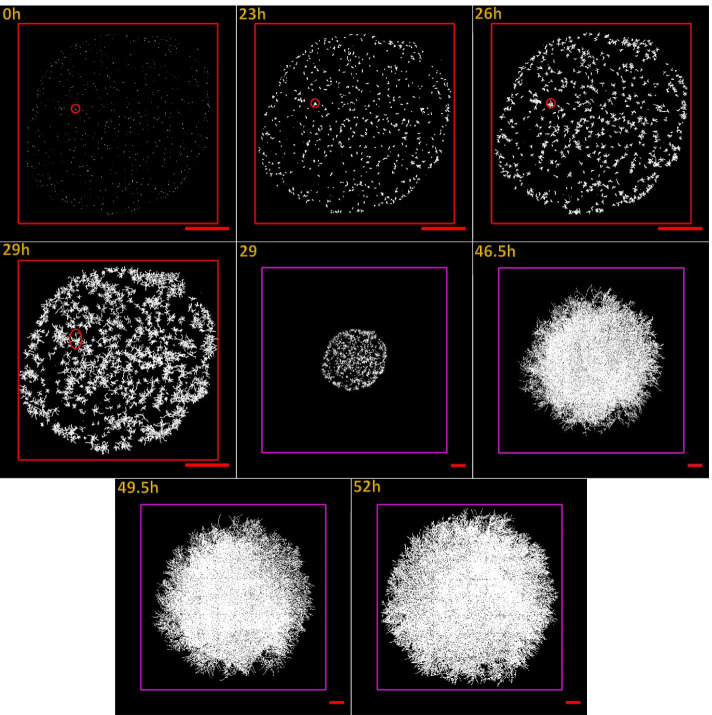
Germination and growth of *P. chrysosporium* on the non-fractionated substrate (culture B). The monitoring was performed at 50X magnification. Images show the growth at time 0, 23, 26,29, 46.5, 49.5 and 52 h. The colour change of the square drawn around the colony from red to purple indicates a change in the scale of the image. The sides of the red square measure 9000 μm. The sides of the purple squares are 25,000 μm long, and the image size is 24,576-pixel × 24,576-pixel. The red circles show the position of a single spore over time. At 23 h, the spore was swollen, and a germ tube was formed. At 26 h, a microcolony was obtained where branching start to take place. At 29 h, the amplification of branching continued, and the tips extended, forming bridges with other microcolonies. One colony can be seen at 46.5 h. The red scale bars measure 2000 μm

The spores were visualized during the early stages of growth (Figs. 2 & 3). The number of spores decreased over time as the spores germinated, and the germ tubes grew into hyphae. The germination rate was determined between 0 and 23 h. The mycelium developed on the surface through tip extension and branching, leading to an interconnected mycelial network and the formation of a colony that grew radially over time (Figs. 2 & 3 red circles).

The fungi growth on the nitrocellulose membrane placed on agar devoid of any miscanthus fraction (culture A) was relatively slow (Fig. 2). The final size of the colony reached 3.9 × 10^7^ μm^2^. The fastest growth (Fig. 3) was observed when non-fractionated miscanthus was incorporated into the agar (culture B). The size of the colony at 52 h was 3.2 ×  10^8^ μm^2^, eight times greater than culture A.

The spore germination percentage and the calculated spore germination rate for the two cultures (Table 1) showed a large difference in their initial growth rates. Culture B, containing the substrate, grew much more rapidly. The percentage of spore germination was greater in culture B, which was reflected in the spore germination rate. The only apparent inconsistency in these observations was in culture A, wherein the germination rate was greater between 23 and 26 h. This observation must be interpreted by considering that the majority of the spore population germinated during this time in culture A.

**Table 1:** The percentage of germinated spores during the first 29 h. All objects observed at time 0 h were considered to be spores. The average size of these objects was 560μm^2^

		Spore germination percentage			Spore germination rate (spore/h)		
	Time (h)	23	26	29	23	26	29
**Substrate**	**A**	17	64	89	5	110	60
	**B**	78	90	94	23	28	11
	**C**	66	90	96	19	51	13
	**D**	64	93	97	18	65	7
	**E**	14	72	81	4	126	18

The deposition of the inoculum droplet containing spores left a marked area on the nitrocellulose membrane, corresponding to the maximum area where germination would first occur. Even after complete absorption of the droplet into the paper, this mark persisted. Colonization of the membrane surface in all experiments occurred first within this marked area. It was shown that the spores near the edge of the mark showed a tendency to first grow inwards rather than outside of the marked area.

### Comparative extent of *P. chrysosporium* growth on substrate fractions

#### Growth as measured by spore germination rate

Observing the percentage spores’ germination, culture A and culture E demonstrated very similar results (Table 1). The growth rate measured in this way was very similar for cultures B, C, and D. The final spore germination percentage was very high for all cultures (81–87%), demonstrating good spore viability. Culture B gave a slightly higher growth rate as measured by this method (Table 1).

#### Growth rates as measured by particle coalescence

At time zero, all objects were considered to be spores. Coalescence was defined as the rate at which the number of objects decreased. The objects consisting of spores and microcolonies (germ tubes and mycelia) were counted at each time point. The variation in the number of objects demonstrated a rapid growth measurement in the early stages of colony development.

**Fig. 4:**
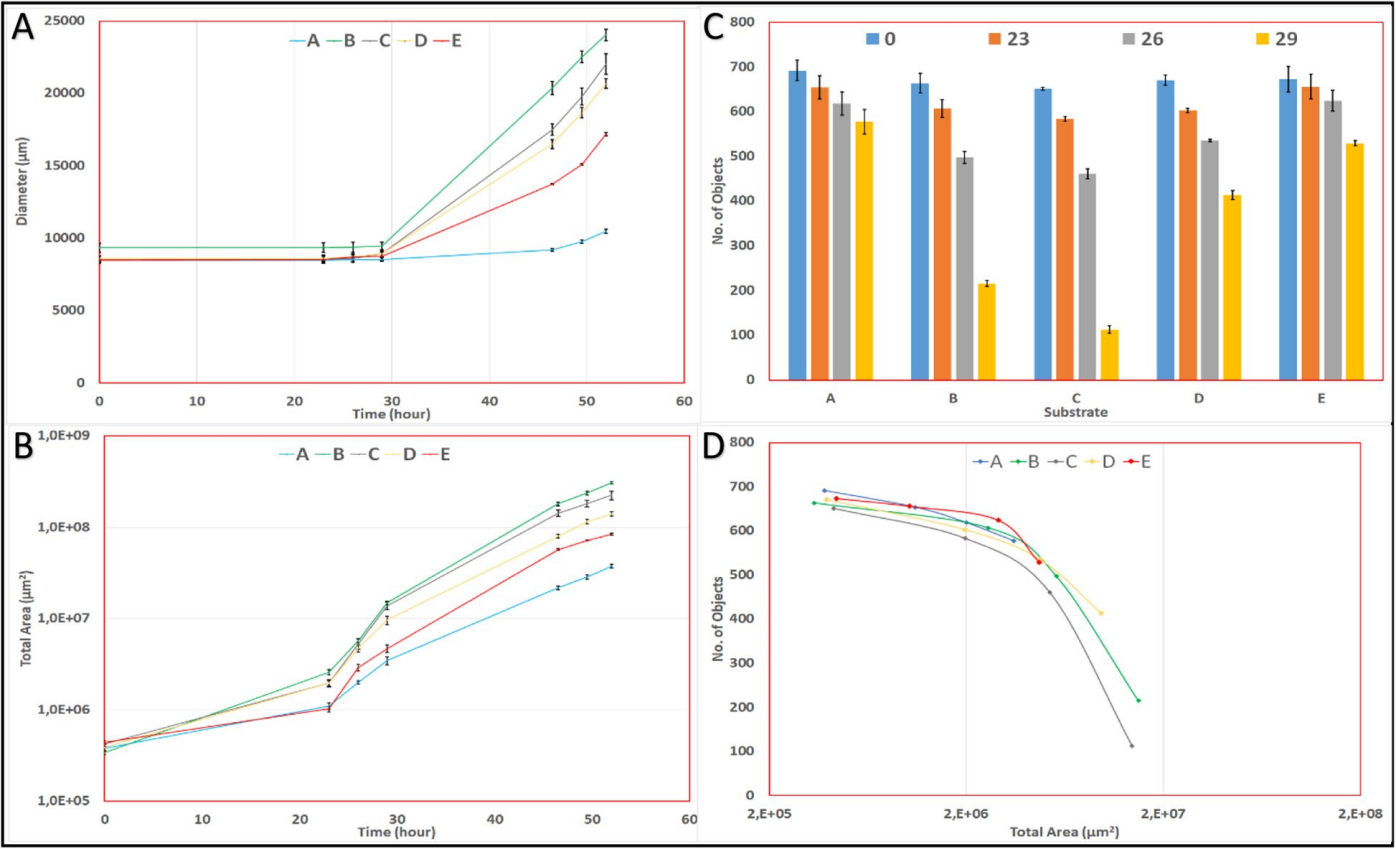
Three types of measurements describe the growth of *P. chrysosporium* on different substrates. Graph (**A**) shows the average colony growth measured by radial expansion. Graph (**B**) shows the average colony growth by surface coverage of the membrane by the mycelium. Histograms (**C**) show the average coalescence patterns of objects; each spore is counted as a single individual particle at time zero. Graph (**D**) shows the average number of objects versus the average total area occupied by *P. chrysosporium*. The vertical error bars in graphs **A**, **B** and **C** indicate the single standard deviation around the average values that have been plotted

The coalescence dynamics were followed for the first 29 h of incubation, after which time it was no longer possible to follow the number of individual objects. The decline in the number of objects accelerated after 26 h, even with the washed solid fraction (Fig. 4 c). The change in the number of objects correlated well with the average size of counted objects (Table 2). At 29 h, the percentage of objects remaining from the initial spores was 85, 30, 18, 60, and 80% on cultures A, B, C, D, and E, respectively. Thus, the fastest growth was observed in culture C, and cultures A and E demonstrated very similar growth rates.

**Table 2:** Average size of counted objects during the growth period 0–29 h. The area is reported in μm^2^; the number of individual objects counted is reported in brackets. After 29 h, it was no longer possible to count the number of objects due to the high degree of coalescence. At the next time point, 46.5 h, there remained 26 individual objects on culture A and just a single object on each of the other cultures. The number of spores deposited on the membrane at time zero varied between 645 and 715, indicating the reproducibility of the inoculation procedure

		Occupied area in μm^**2**^, (no. of objects)			
	Time (h)	0	23	26	29
**Culture**	**A**	5.64 × 10^2^ (715)	1.76 × 10^3^ (680)	3.23 × 10^3^ (645)	6.35 × 10^3^ (605)
	**B**	5.20 × 10^2^ (687)	4.39 × 10^3^ (627)	1.24 × 10^4^ (485)	7.48 × 10^4^ (209)
	**C**	6.64 × 10^2^ (648)	3.65 × 10^3^ (588)	1.26 × 10^4^ (472)	1.27 × 10^5^ (120)
	**D**	5.83 × 10^2^ (660)	3.10 × 10^3^ (598)	8.12 × 10^3^ (533)	2.04 × 10^4^ (424)
	**E**	6.48 × 10^2^ (645)	1.53 × 10^3^ (628)	4.42 × 10^3^ (601)	8.11 × 10^3^ (524)

#### Growth rates as measured by radial extension

Once complete coalescence had occurred, the mycelial mat could be considered a single colony. The radial extension of the colony was the internal distance between the circumferences of each colony.

Due to the observation that initial growth took place within the marked area where the spores were deposited, no radial growth was observed during the first 29 h. After this period, the colony diameters for cultures B and C increased linearly but accelerated for the other cultures (Fig. 4a). After 29 h, the fastest growth was observed in culture B. The fungus was growing considerably slower on culture E than cultures B-D, and finally, the lowest radial expansion was registered on culture A (Table 3).

**Table 3:** Occupation rate (μ) on different substrates. The values of the mean extension rate (v) of the P. chrysosporium colony are correlated with the growth measured by surface area occupation for the same time intervals. The coefficient of determination (R^2^) was calculated for each regression line

Growth rate	Occupied area				Radial expansion	
	23 h to 29 h (3 points)		29 h to 52 h (4 points)		29 h to 52 h (4 points)	
Culture	μ_1_ (h^−1^) exponential fit	R^2^	μ_2_ (h^−1^) exponential fit	R^2^	*v* (μm/h) linear fit	R^2^
A	0.195	0.999	0.101	0.999	64	0.7
B	0.290	0.997	0.134	0.995	585	0.98
C	0.326	0.999	0.124	0.996	502	0.99
D	0.257	0.997	0.121	0.998	398	0.93
E	0.248	0.957	0.133	0.989	312	0.96

#### Growth rates as measured by surface colonization

Image analysis allowed for the determination of the total surface occupied by the spores and mycelia at any given time. This total area consisted of the sum of all the white pixels present on the image. The inferred growth is related specifically to the superficial growth area in the marked surface.

Growth measured in terms of the occupied area was exponential during two distinct periods; 23–29 h and 29–52 h (Fig. 4b & Table 3). Between 23 h and 29 h, the exponential growth rate of *P. chrysosporium* in culture C was the highest (0.326 h^− 1^), followed, in order, by cultures B, D, E, and the agar control (culture A) (Table 3). The growth rates of cultures D and E were very similar.

During the second period (29–52 h), the ranking from high to low culture growth rates was B, E, C, D, and A (Fig. 4b). The growth rates of cultures B and E were very similar, and C and D were also similar. Culture E showed a rapid increase in growth during this phase compared to all other cultures (Table 3).

**Fig. 5:**
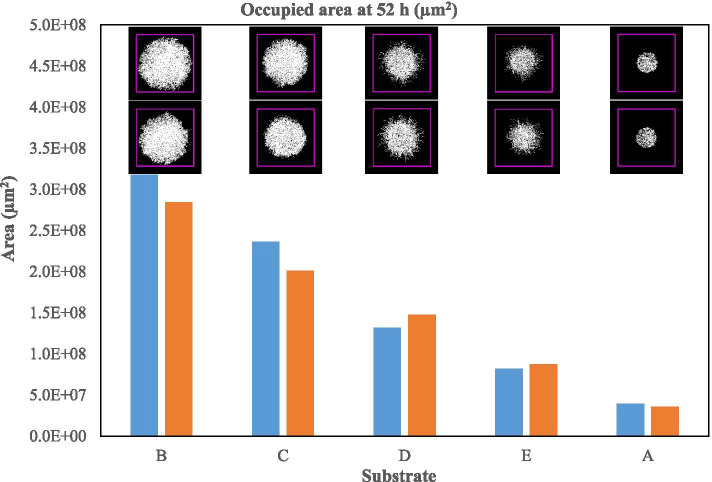
The area occupied by *P. chrysosporium* mycelia after 52 h. The blue and orange bars indicate the data of duplicate cultures for each substrate. The corresponding images visually represent the growth and morphology of the colonies at each time point. The upper row of images corresponds to the data represented by the blue bars, and the lower images correspond to the data represented by the orange bars. Each side of the purple square is 25,000 μm

The mycelium in culture B covered 3.0 ± 0.2 × 10^8^μm^2^, 8-fold greater than that for the negative control (culture A). At the same time point, culture C was 78% of culture B; culture D was 42% of culture B, and culture E was 26% of culture B. The use of non-fractionated miscanthus (Fig. 5) resulted in the fastest growth of the organism followed by the soluble fraction (culture C), the unwashed solid fraction (culture D), and the washed solid fraction (culture E). If the growth area on agar alone (culture A) is subtracted from all growth data, the sum of the growth measured on cultures C and D provided a value close to that of culture B. Thus, the covered area on culture C at 52 h represented 64% of the covered area of culture B; the soluble fraction is largely responsible for the extent of mycelial growth.

#### Chemical analysis of solid and soluble fractions of pre-treated miscanthus by FTIR

FTIR spectroscopy of the multiple miscanthus fractions used for *P. chrysosporium* growth provided rapid semi-quantitative information on the functional groups present, and therefore, on the polymers and molecules potentially available for fungal growth within the substrates. All the substrates in cultures B to E were analysed. The correspondence between the name of the FTIR sample and the type of the substrate from which it is originated is described in Methods. The main functional groups were assigned to cellulose (C), hemicellulose (HC) and lignin (L) in FTIR spectra according to the literature [73–85].

The FTIR spectra of substrates used in cultures B, D, and E are almost identical, suggesting few changes in the relative content of lignin, hemicellulose, and cellulose due to the pre-treatment. All these substrates contained solid fractions. Results for samples C and Ew were similar but different from samples B, D, and E, especially for the functional groups related to xylan (1734 cm^− 1^) and aromatic compounds (1605 cm^− 1^). The soluble material remaining in substrate D was similar to substrate C (Table 4, Additional file 1: Supplementary Figure 1), demonstrating that the liquid fraction is also present in the unwashed solids fraction.

**Table 4:** Selected absorption band data which correspond to the wavelength of the spectra for the solid and soluble fractions. Abbreviations: cellulose (C), hemicellulose (HC), and lignin (L)

Functional groups		HC	L	L	L	L-C	C-HC	C	C-HC		L
Wavenumbers (cm-1)		1734	1605	1514	1463	1427	1376	1322	1055	1036	896
Samples & absorbance	FTIR-C C1	0.2	0.6	0.2	0.2	0.2	0.2	0.8	0.8	0.7	0.0
	FTIR-Ew ^C2^	0.2	0.6	0.2	0.2	0.2	0.2	0.7	0.7	0.7	0.0
	FTIR-B	0.5	0.5	0.3	0.3	0.3	0.3	1.3	1.3	1.2	0.1
	FTIR-D	0.6	0.5	0.3	0.3	0.3	0.3	1.2	1.2	1.2	0.2
	FTIR-E	0.5	0.5	0.3	0.3	0.3	0.3	1.2	1.2	1.2	0.1

^C1^ represents 1.5% and ^C2^ 0.25% of the total substrate in culture B. The main differences between the fractions are highlighted in black or grey

The elemental (CHNSO) analysis of miscanthus, agar, and the nitrocellulose membrane showed the total absence of nitrogen and sulphur in miscanthus, while the two elements existed in the nitrocellulose membrane (Table 5).

**Table 5:** Elemental analysis of untreated miscanthus, agar, and the nitrocellulose membrane. The average percentage by mass of each element of total CHNSO is represented for duplicates of samples at 0% moisture

CHNOS composition	% C	% H	% N	% S	% O
Element of device					
*Miscanthus x giganteus*	45.9 ± 1.6	6.0 ± 0.2	0.0	0.0	48.1 ± 1.8
Nitrocellulose membrane	26.3 ± 1.9	2.6 ± 0.5	11.0 ± 1.0	0.6 ± 0.1	59.4 ± 3.6
Agar	42.7 ± 0.2	6.4 ± 0.1	0.0	0.9 ± 0.0	50.0 ± 0.3

## Discussion

The main challenge in estimating the growth and morphological development of *P. chrysosporium* by non-destructive microscopic observations was to develop a method for estimating the fungal biomass on natural lignocellulosic biomass and its fractions. Autoclaving miscanthus in aqueous suspension gave two fractions (soluble and solid) through physical separation. Growth of *P. chrysosporium* on each fraction of the biomass was measured using four different methods: 1) the spore germination rate, 2) the coalescence rate, 3) the radial expansion of the mycelial colony, and 4) the surface occupation wherein the actual surface covered by the mycelium was calculated. Of these methods, surface occupation proved to be the best method because it provided information about *P. chrysosporium* growth continually throughout the incubation period*.* The rate of surface occupation was used to measure fungal growth on each substrate fraction. Other researchers have reported the radial growth of brown rot fungi *Postia placenta* at a constant rate on the agar surface and concomitant fungal growth into the agar [59, 86]. The latter was directly related to the average growth rate of the tips [87].

The substrates studied were the non-fractionated (B), the soluble (C), the unwashed solid (D), and washed solid (E) fractions. Substrate (B) differed from the other fractions in that it was sterilized in the agar medium, whereas, the other fractions were sterilized in aqueous suspensions and mixed with the agar solution post sterilization. The application of an inverted grey nitrocellulose membrane to support spore deposition, germination, and mycelial growth was the particular innovation that allowed good image acquisition in a non-destructive manner. The porous membrane allowed diffusion of water and nutrients to the spores and the developing mycelium while acting as a physical barrier that prevented observable mixing of the organism and its substrate. Any possible enzymes would have diffused in the opposite direction to contact the substrate. The success of this experimental protocol was evident from the high spore viability observed.

The experimental system provided good contrast for the visualization of the mycelia, facilitating image acquisition and analysis. Spore adhesion to the surface was strong enough to maintain their original positions throughout growth (Figs. 2 & 3).

Agar was the common ingredient in all cultures, principally to supply moisture throughout mycelial development while keeping the membrane flat. The agar also acted as the carrier for the substrate, although weak fungal growth on agar (A) without any additional substrate was observed (Fig. 2). Agar-degrading bacteria have been previously reported in the literature [88], capable of growing on agar while utilizing it as the sole nutrient source. Separate experiments (data not shown) showed weak growth on agar alone without the nitrocellulose membrane. The decomposition of agar ingredients (water-soluble polysaccharide) during sterilization could provide carbon and nitrogen sources for spore germination [89, 90], enabling this low level of growth.

The area colonized by the mycelia after 52 h depended on the substrate present in the agar under the membrane (Fig. 5). The average areas covered by *P. chrysosporium* containing any of the substrates tested (B to E fractions) were, without exception, larger than the agar control (culture A). The fastest overall growth rate was obtained on the non-fractionated substrate B (culture B). Although the early growth rate was faster with the soluble fraction alone (culture C), growth on the non-fractionated substrate accelerated and by 52 h had surpassed the extent of growth on the soluble fraction (culture C). *Neurospora crassa, Arthrobotrys oligospora* and *Trichoderma reesei* showed different growth behaviours on PDA (Potato Dextrose Agar) and LMN (Low Nutrient Medium) [56]. *Neurospora crassa* colonized the entire PDA medium zone in less time (< 24 h) than on LMN (40 h). It generated more hyphal tips, and its mycelial network was denser on PDA. All strains showed the same performance on LMN, but not on PDA [56].

Generally, growth on the unfractionated substrate B and the soluble extract C were similar. Growth on the washed and unwashed substrates (E and D) was also similar, but the growth profiles of the two cultures (B&C vs. D&E) were different. Growth on the unwashed substrate (D) was initially relatively fast compared to that on washed solids (E). Initially, the growth in culture E resembled that of the agar control culture (A). On the other hand, once growth on the miscanthus solid fractions began, it proceeded rapidly (Fig. 4b). This observation suggests that the soluble fraction is initially more available to the fungus but that once the solid fraction becomes available due to enzyme production, the rate of its assimilation is similar to that of the soluble fraction. The growth on the solid fraction can be quite slow initially but then accelerates once growth is established. This pattern is most evident from the growth speed measured on the washed solids (culture E). When available, the soluble and the solid components of the substrate are consumed at the same time, but the easily assimilable component of the soluble fraction can prompt a fast-initial growth rate. Similarly, the simultaneous consumption of both soluble and insoluble components on wheat straw by *Ganoderma lucidium* has been reported [91].

After 52 h, the growth rate observed on complete substrate could be reproduced arithmetically by the sum of the growth rates on cultures C and D (Table 6). The additivity started after 46.5 h (Fig. 4b).

**Table 6:** Additive effect of different substrate fractions on *P. chrysosporium* growth at 52 h

Calculation	B-A	C-A	D-A	E-A	(C-A) + (D-A)	(C-A) + (E-A)
**Occupied area** **(μm**^**2**^ **× 10**^**8**^**)**	2.6	1.8	1.0	0.4	2.8	2.3

B-A C-A, D-A, E-A: correspond to the mean occupied area on the defined substrate (B, C, D, E) minus the mean occupied area on the substrate (A). (C-A) + (D-A): The addition of the mean occupied area on the soluble fraction (Substrate C) and that on the unwashed solid (Substrate D) after the subtraction of the mean occupied area on agar (Substrate A) from each culture

Microbial growth is conventionally characterized by the exponential rate of growth (μ) and is indicated as per unit time. This growth is a species- and condition-dependent constant value and can be used in conjunction with Monod’s equation to model microbial growth [92]. The determination of mycelial growth and fungal biomass formation is rather challenging due to the close association of the fungal biomass with the substrate. As the former increases, the latter decreases, rendering biomass determination difficult.

The mycelium tends to fill the initial area where the spores are deposited; therefore, early growth is not captured by radial colony expansion. After 29 h of incubation, the growth of *P. chrysosporium* could be satisfactorily followed either by surface occupation or radial growth rates (Fig. 4 & Table 3). We have demonstrated that colony surface colonization, as determined by microscopy and image analysis, is a suitable method for following mycelial growth non-destructively but was limited by the growth of the mycelium in 3D. The determination of the very early growth rate is of particular interest as growth can be considered free of any restrictions at this early stage. Coalescence in time proved useful for measuring the initial growth rate, but image analysis in this method was very labour-intensive and was limited by the coalescence of objects within the initial droplet area.

The observations in this work are consistent with the hypothesis that *P. chrysosporium* initially consumes the soluble fraction of miscanthus while it produces the metabolic machinery required to degrade the solid fraction. Once hydrolytic and oxidative enzymes are secreted, the solid fraction could be digested, and the mycelium occupied the surface at an even faster rate by efficient co-utilization of the two fractions. The co-utilization of soluble and insoluble nutrients of wheat straw has been reported to result in the highest fungal growth and laccase production by *Ganoderma lucidium* [91].

In our study, growth was measured in 2D; therefore, the penetration of the mycelium through the membrane and into the agar could not be observed. It has been shown that hyphae, in direct contact with a polyester (PET) membrane, penetrate membrane pores (3 μm), grow through the membrane, and emerge on the other side [93].

In our experiments, *P. chrysosporium* was not able to grow on moist nitrocellulose membrane even after 52 h (data not shown) in the absence of agar. The absence of readily available sources of carbon does not allow the invasion of the nitrocellulose support by the organism. We propose that the soluble nutrients can impregnate the support and thus feed the spores on the side that is exposed to the air.

The FTIR data analysis showed that the soluble molecules (sample FTIR-Ew) associated with the solid fractions of the substrate had the same composition as the soluble fraction (Sample FTIR-C) (Table 4 & Additional file 1: Supplementary Figure 1). Both contained lignin and carbohydrates, which means that substrate D contained two sub-fractions: the easily assimilated soluble fraction and the physically associated solid fraction. The latter was initially more difficult to digest.

The FTIR data are consistent with what is generally observed after autoclaving lignocellulosic material. In mild hot water pre-treatment, it can be supposed that limited hydrolysis and rearrangement of hemicelluloses and lignin occurred [94], leading to the release of small amounts of acid-soluble lignin, oligosaccharides, monomer sugars (xylose, glucose) and other extractives (phenolic acids, aliphatic esters, etc.) into the liquid phase [95, 96]. The soluble components were demonstrated to be good substrates for boosting the initial growth of the *P. chrysosporium* under our experimental conditions.

Elemental analysis of C, H, N, S, and O (Table 5) showed a total absence of nitrogen (0%) in miscanthus and agar, but it was present at 11% (w/w) in the nitrocellulose membrane. Sulphur was not detected in miscanthus but was in the agar and nitrocellulose membrane at 0.9 and 0.5%, respectively. The liquid fraction of miscanthus obtained by a mechanical press contained 0.8% N and 1% S as well as other minerals [97]. Agar is composed of carbon, hydrogen, oxygen, and sulphur [98]. Although the growth of the organisms on nitrocellulose alone is very slow [99] and non-existent on the time scale of the experiment (data not shown), we propose that *P. chrysosporium* grows using the carbon and nitrogen sources either present in the treated miscanthus or small degradation products of the agar. Nitrogen content was suggested to play a crucial role in mediating the growth of *P. chrysosporium*. and the production of lignocellulolytic enzymes [100]. A high C/N ratio is generally assumed to offer the best growth and activities of LiP and MnP [101, 102]. Under nitrogen-poor conditions, it is supposed that *P. chrysosporium* recycles nitrogen in its cells [101, 102]. Under these circumstances, the elemental nitrogen from the nitrocellulose membrane could be utilized with the carbon from the miscanthus.

## Conclusions

An efficient method based on microscopy and image analysis was developed to follow and quantify *P. chrysosporium* growth on miscanthus fractions, and our results showed growth differences of the microorganism on the different fractions.

*Phanerochaete chrysosporium* was able to grow weakly on agar alone as a substrate, and after correcting the results for this growth, the surface occupation was used as a measure of growth. The coalescence dynamic as measurement could offer a new rapid method for early growth analysis. The methods used in this study were limited to 2D observation on physical support, which prevented the detailed analysis of the real fungal biomass such as the volume of mycelium in voxels and its behaviour in direct contact with the natural solid lignocellulosic biomass.

Different phases of growth could be identified. From 0 to 23 h, the spores germinated and occupied the area of the droplet initially placed on the membrane. During this phase, growth was fastest on the soluble fraction of miscanthus. The presence of washed solids did not influence the growth rate of the organisms compared with agar alone, so the solid fraction was not immediately attacked. A second growth phase between 23 and 29 h showed a rapid acceleration of all growth rates. Finally, during the last growth phase, the cultures containing solid fractions overtook the growth of culture that contained only the soluble fraction. This leads us to conclude that degradation of the solid fraction by *P. chrysosporium* does not pose a problem to the organism, and once the enzymic machinery is in place, the organism can grow even more rapidly on the solid fraction.

This method for measurement of fungal growth may be applied to other studies to help further our understanding of the mechanism in which the fungi attack their substrate. The study reported here should be followed by a particular focus on the temporal production of the enzymes, such as cellulases (β-glucosidases, endoglucanases, cellobiohydrolases), hemicellulases (endo-1,4-β-xylanases, xylan 1,4-β-xylosidases), and ligninases (manganese peroxidases, lignin peroxidases).

## Methods

### Materials

Commercial *Miscanthus x giganteus*  from AGROMI SAS (Pauvres, France) was used as a lignocellulosic substrate. It is a common mulching material, composed exclusively of miscanthus. The aerial parts of the plant were harvested at the end of March 2017, shredded and dried before conditioning. The C/N (w/w) ratio was 100:1 (supplier information) with a moisture content of 15% (w/w), determined by halogen lamp moisture analyzer OHAUS™ MB-45. The stems were milled sequentially to 1 mm (Retsch Cross Beater Mill SK1) and sifted through sieves of mesh sizes 4 mm, 2 mm, and 1 mm. The particles were pulverized first to 200 μm and then to 80 μm (Ultra Centrifugal Mill ZM 200; Retsch®). Ring sieve cassettes with 0.2- and 0.08-mm conical holes were used for this purpose. The 80 μm particles were subsequently selected for the experiments.

*Phanerochaete chrysosporium* (BRFM 531) was obtained from the CIRM-INRAE collection (https://www6.inrae.fr/cirm/) in sterile glycerol solution (10% v/v) and stored at 4 °C.

Agar Sigma^©^ (Sigma-Aldrich A1296 Lot # BCBR4069V) was included in all solid growth media before pouring the molten preparations in Petri dishes (Thermo Scientific™ Sterilin™ Ø = 55 mm; ht. = 12 mm). Inverted grey gridded cellulose nitrate membrane filters (Sartorius™ 13,006--47----ACN, Ø = 47 mm; pore size: 0.45 μm) were used as support for the spore suspension and subsequent growth of *P. chrysosporium.*

Zeiss Microscope (Axioplan 2, Carl Zeiss Microscopy GmbH, Germany) Imaging was used with a Malassez counting chamber for estimation of spore concentration in suspensions. Observations of growth in all experiments were performed using an automated microscope (Microscope Zeiss Axio Zoom V.16).

### Inoculum preparation

*Phanerochaete chrysosporium* was grown in Petri dish culture on MAE medium containing (w/v): malt extract (2%) (Sigma-Aldrich 70,167 Lot # BCBR6119V) and agar (2%) (Sigma-Aldrich A1296 Lot # BCBR4069V). The strain was kept in perpetual culture with twice weekly sub-culturing on solid media.

To prepare a spore suspension, a fragment (5 mm Ø) of mycelial growth was cut from the margin of an actively growing colony and incubated on fresh growth medium at 25 °C for 14 days. The spores of *P. chrysosporium* were detached and harvested by using seven sterile glass beads and sterile Milli-Q water (5 ml) containing 0.2% (v/v) Tween 80 detergent. The conidial suspension was recovered, and the spore density was estimated by microscopy. The suspension was diluted with sterile Milli-Q water to a concentration of 100 conidia μl^− 1^ and stored for 67 ± 2 h at 4 °C.

### Experimental procedure for growth on fractionated biomass

**Fig. 6:**
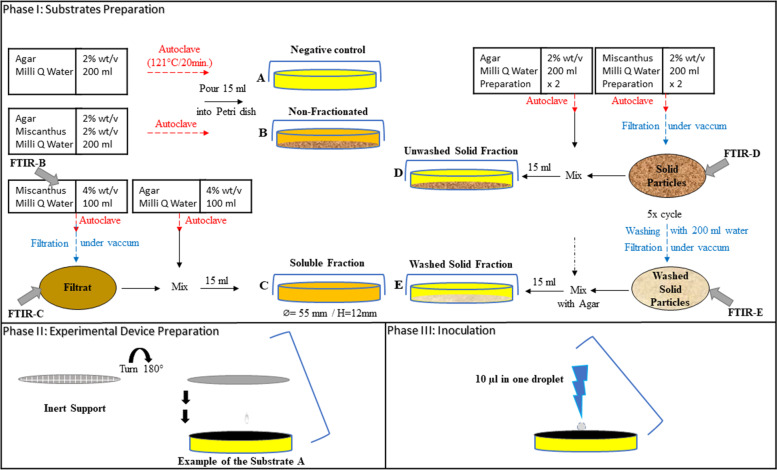
Experimental apparatus and procedure. Phase (I) shows the preparation of all growth media. Phase (II) demonstrates the experimental device. The grey nitrocellulose membrane was placed, grid side down, on the surface of the agar containing different substrates to hide the white lines and provide a homogeneous background for imagery. The preparations were tempered to 70 °C

Miscanthus was sterilized at 121 °C for 20mins (which also acted as a hydrothermal pre-treatment of the biomass), followed by tempered at 70 °C for 15mins in a water bath. The growth media were prepared using agar containing the pre-treated unfractionated substrate (miscanthus; 80 μm) or its fractions (Fig. 6). All agar gels were covered with a filter membrane that was used as a support for the growth of the mycelium. Growth of *P. chrysosporium* was monitored on duplicate cultures on five different culture media. All cultures were incubated at 25 °C.

i. **Non fractionated (B) and control (A) substrates**

The agar was included primarily to provide moisture during incubation. The negative control (culture A) contained only agar (2% w/v), on which no fungal growth was expected.

The non-fractionated substrate, used for culture B, contained miscanthus (4 g), agar (4 g), and Milli-Q water (200 ml) (Fig. 6). After sterilization, 15 ml of well-mixed medium were poured into each Petri dish (55 mm diameter) and allowed to solidify (Fig. 6). This culture was included to provide a reference point for the natural growth of the organism.

ii. **Fractionated substrates**

Miscanthus (4 g) and agar (4 g) were autoclaved separately in Milli-Q water (200 ml each). Using the former suspension, the soluble and solid fractions were separated by sterile filtration under vacuum (Fig. 6). The filtrate (substrate C) was mixed with an equal volume of agar solution and poured into the Petri dish. This substrate was used for the growth of culture C that contained the “soluble miscanthus fraction.” The solid portions were added into an agar solution (200 ml) and dispensed (15 ml) into Petri dishes. The substrate designated as “unwashed solid fraction” consisted of solid particles that remained on the filter after simple filtration of the autoclaved miscanthus suspension.

This fraction was used for culture D. In a separate preparation, the solid fraction obtained after filtration was washed with sterile Milli-Q water (1 L) to form the “washed solid fraction,” which after addition into the agar solution was called substrate E and was used for culture E (Table 6).

iii. **Experimental system and culture procedure**

A drop of spore suspension (10 μl) was deposited onto the centre of the filter. After 10 minutes (the time for the drop to be absorbed into the paper), the plates were sealed and observed by microscope (designated time 0). All cultures were prepared and incubated in duplicate. The average data value is presented in results.

### Microscopy

Growth was monitored using a 50X/3.3 REO objective lens and a Hamamatsu Camera. The unopened Petri dish was placed on a motorized stage (with X-Y coordinates). A brightfield configuration with a bespoke image acquisition process (ZEN blue edition software) was used with 100% illumination and 300 ms exposure time per image. The number of tiles used for imaging was chosen depending on the size of the entire colony. The overlap between two neighbouring tiles was 10%. The one-pixel size was equivalent to 1.3 x 1.3 (µm^2^), 2, and the depth of focus was 15.6 μm.

### Image processing and measurements

2D composed images were exported in TIFF format and processed using ImageJ™ and reassembled with the “Grid/Collection stitching” plugin [103]. The median filter was used to minimize the variation in the grey values of image pixels within a specific neighbourhood. The “Enhance Local Contrast (CLAHE)” was used to enhance the local contrast. The unevenly illuminated background was corrected by the “subtract background” command. Image processing continued using a combination of median, mean, and maxi filters to improve the processing and reduce noise. Finally, the 8-bit image was converted to a binary image. “Yen thresholding” was used for binarisation of images acquired at time 0, whereas the “Otsu threshold” method was applied for all subsequent images. The image toolbox of Matlab measured colony diameter, the area occupied by the mycelium, the total number of objects (spores and later, independent networks), and their areas. The spores and colonies (germ tubes, mycelia generated from individual spores, mycelium resulting from the 2D visual connection of many mycelia) are supposed to appear as discrete objects in binary images. As the stage holding the specimen was adapted to move in both X and Y directions only, the measurements were performed pixel by pixel on the surface.

### Elemental analysis

The samples to be analysed were lyophilised (4.10^− 5^ bar pressure for 72 h), and 1 mg duplicates were used. The elemental (C, H, N, S, and O) composition was determined for milled miscanthus biomass, agar powder, and the nitrocellulose membrane using a CHNS-O analyser (Thermo Scientific Flash 2000 Organic Elemental Analyzer, USA) and the “Eager Xperience” software (Thermo Scientific).

### Description of FTIR (Fourier transform infrared) sample analysis

All substrates (B to E) were lyophilised before preparing the samples for FTIR-analysis (Fig. 6; Table 7).

**Table 7:** Culture media and respective substrates used; corresponding denomination relative to FTIR analysis

Culture	Substrate type and designation	Identification of the freeze-dried substrate analysed by FTIR
A	Negative Control No lignocellulosic biomass	–
B	Non-Fractionated Substrate substrate B	FTIR-B
C	Soluble Fraction - substrate C	FTIR-C
D	Unwashed Solid Fraction substrate D	FTIR-D
E	Washed Solid Fraction - substrate E	FTIR-E

In addition to the substrate fractions, FTIR was used to analyse the “FTIR-Ew” samples obtained by the lyophilisation of the wash-water recovered from washing substrate D (Table 6).

### Sample conditioning and FTIR acquisition

Lyophilised dry matter (2.4 mg) was mixed and ground with 200 mg KBr to form disk samples. The spectra of all samples were collected in transmittance mode by the FT-IR spectrometer (Thermo Fisher Nicolet, 6700 FTIR). All spectra were acquired from 4000 to 400 cm^− 1^ at 4cm^-^^1^ intervals, and the absorbances measured were averaged over 16 scans and corrected by background-air subtraction. The individual spectra were corrected for their baselines and normalized to the same total area using “OMNIC” (Thermo Fisher) version 8 software. The intensity of the absorption characteristic for each functional group of interest was then determined as the height of the signal at the relevant wavenumber. For data recovery and representation (Additional file 1: Supplementary Figure 1), SpectraGryph v1.2.12 software (Optical Spectroscopy, Germany) was used.

## Supplementary Information

**Additional file 1: Supplementary Figure 1.** FTIR absorption spectra for the solid (A) and soluble (B) of pretreated *Miscanthus x giganteus* fractions. Abbreviations: cellulose (C), hemicellulose (HC), and lignin (L).

## Data Availability

The materials and methods section are sufficiently detailed for the experiments to be repeated. The datasets used and/or analysed during the current study are available from the corresponding author on reasonable request.

## Abbreviations

*P. chrysosporium*: *Phanerochaete chrysosporium*
*M. giganteus*: *Miscanthus x giganteus*
SSF: Solid state fermentation
FTIR: Fourier-transform infrared spectroscopy
NC: Nitrocellulose
PDA: Potato Dextrose Agar
LMN: Low Nutrient Medium
2D: Two dimensional
3D: Three dimensional
L: Lignin
C: Cellulose
HC: Hemicelluloses
