# Supplementary material for: Morphological growth pattern of Phanerochaete chrysosporium cultivated on different Miscanthus x giganteus biomass fractions
Source: BMC Microbiol. 2021 Nov 17;21:318. doi: 10.1186/s12866-021-02350-8 (PMC8597199; doi:10.1186/s12866-021-02350-8)

**Additional file 1**

**Supplementary Figure**

**FTIR absorption spectra for the solid and soluble pretreated *Miscanthus x giganteus* fractions**

Hassan KHALIL^a,c^, Estelle LEGIN^c^, Bernard KUREK^c^, Patrick PERRE^a,b^, and Behnam TAIDI^a,b^*

**Supplementary Figure 1 : FTIR absorption spectra for the solid (A) and soluble (B) pretreated *Miscanthus x giganteus* fractions.** Abbreviations: cellulose (C), hemicellulose (HC), and lignin (L).


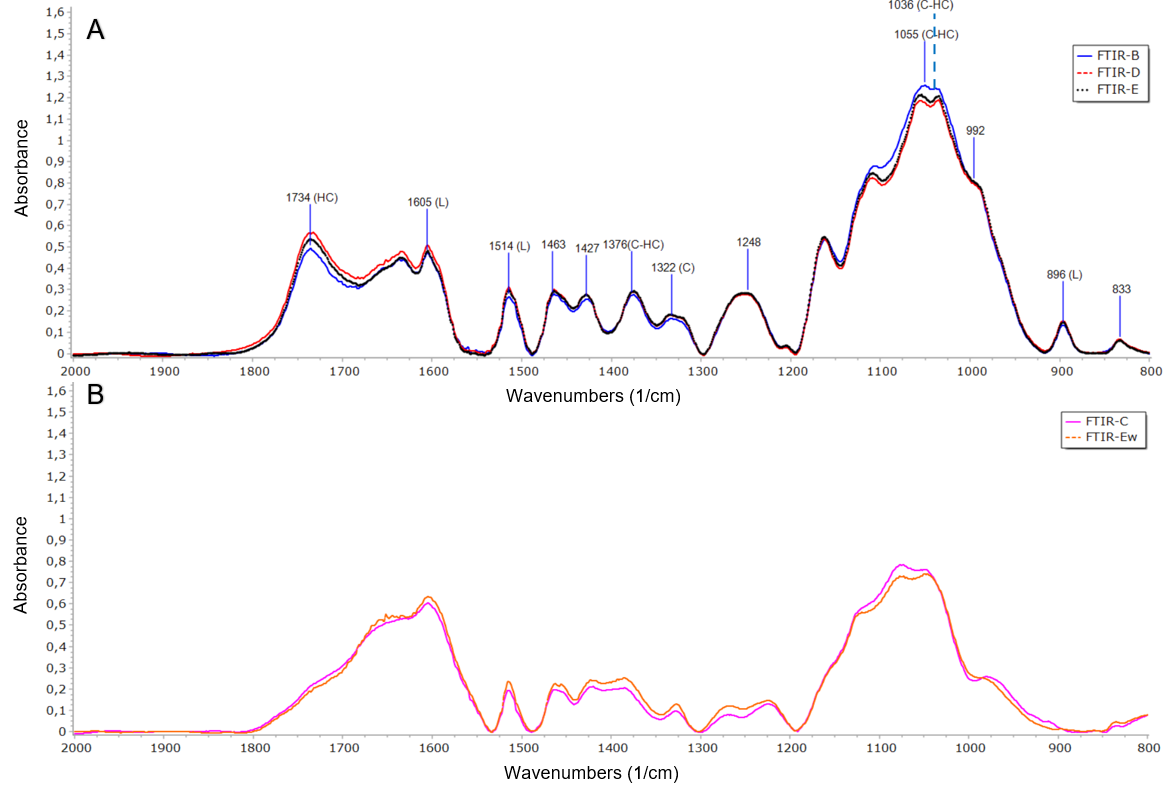

Supplement: Supplementary file 1 — Additional file 1: Supplementary Figure 1. FTIR absorption spectra for the solid (A) and soluble (B) of pretreated Miscanthus x giganteus fractions. Abbreviations: cellulose (C), hemicellulose (HC), and lignin (L). [file 12866_2021_2350_MOESM1_ESM.docx]
